# The Role of *Acsl1* and *Aldh2* in the Increased Risk for Liver Cancer in Offspring of Obese Mothers

**DOI:** 10.3389/fmed.2022.907028

**Published:** 2022-06-27

**Authors:** Beat Moeckli, Stéphanie Lacotte, Christian Toso

**Affiliations:** ^1^Division of Visceral Surgery, Department of Surgery, Geneva University Hospitals, Geneva, Switzerland; ^2^Hepatology and Transplantation Laboratory, Division of Visceral Surgery, Department of Surgery, Faculty of Medicine, University of Geneva, Geneva, Switzerland

**Keywords:** hepatocellular carcinoma, maternal obesity, origin of disease and health, microbiota, epigenetics

## Introduction

The obesity rate continues to increase and already exceeds 30% in many regions of the world (1). This truly global phenomenon affects women of childbearing age particularly and thus more and more children are born to obese mothers every year (2). Large-scale epidemiological studies have shown that maternal obesity has deleterious effects on offspring health such as increased risk for obesity, liver steatosis and even certain types of cancers (3–5).

## The Role of *Acsl1* and *Aldh2* in Maternal Obesity Related Liver Disease

In a recent study, Sun et al. showed in a murine model of hepatocellular carcinoma that offspring of obese mothers display an increased risk to develop liver cancer (6). Indeed, the authors observed three times as many large tumors in offspring of obese mothers compared to offspring of mothers fed a normal diet. The authors propose a mechanistic link between maternal high-fat diet and the development of liver cancer through the downregulation of two genes, Acyl-CoA synthetase long chain family member 1 (*Acsl1*) and Aldehyde dehydrogenase family member 2 (*Aldh2*). *Acsl1* codes for an enzyme that is implicated in lipid synthesis and fatty acid degradation through the conversion of free long-chain fatty acids into fatty acyl-CoA esters, the gene is located on 4q35.1 (7). *Aldh2* encodes an enzyme of the major oxidative pathway of alcohol metabolism, its genomic location is 12q24.12 (8). Both genes are involved in metabolic processes of the liver. The study suggests that a gradual downregulation of these two genes over several generations is mediated through a specific microRNA, miR-27a-3p.

**Figure 1:**
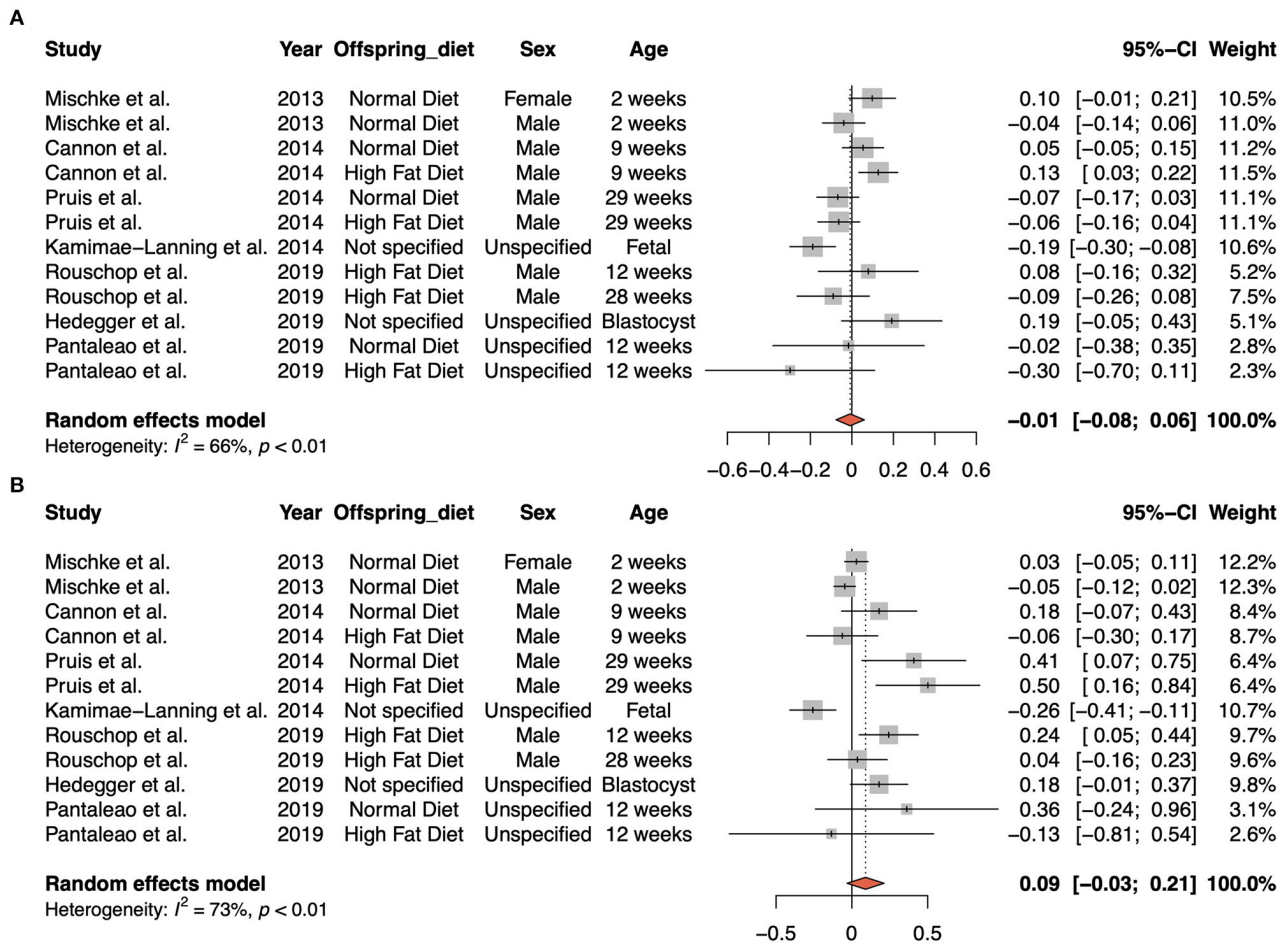
Meta-analysis of expression of *Aldh2* and *Acsl1* in offspring of obese mothers in mouse studies. The upper panel represents the meta-analysis of *Aldh2* **(A)** and the lower panel *Acsl1* **(B)**. A negative value in the plot indicates a downregulation and a positive value and upregulation of the respective gene.

We recently analyzed the impact of maternal obesity on the gene expression profiles in the offspring (9). We compiled 11 previously published datasets that compared the gene expression in offspring from obese and non-obese parents. All selected studies were performed in mice and assessed the gene expression in the liver of first-generation offspring of different age. We identified a number of genes and pathways that were consistently dysregulated (10–15). In this context, we assessed the gene expression level of *Acsl1* and *Aldh2* in these datasets. In contrast to the results of Sun et al. we did not see any overall changes in expression for these genes (Figure 1). For a number of datasets *Acsl1* is even significantly upregulated. Based on this meta-analysis data we can confidently state that maternal obesity does not affect the expression of *Acsl1* and *Aldh2* in the first generation of obese offspring.

The results about the increased risk for liver cancer in the offspring of obese mothers described by Sun et al. is convincing and in line with epidemiological data (3, 5). A downregulation of the two proposed genes may well play a role in the experimental setting of the authors. However, we believe it is unlikely that these results are translatable to different experimental contexts or the clinical reality. A regulation of these metabolic genes is unlikely to be solely responsible for the increased risk of liver cancer in offspring of obese mothers.

## Discussion

The liver is in close communication with the intestinal tract and exposed to a considerable amount of bacterial products and metabolites. A dysbiosis of the gut microbiome can promote the development of hepatocellular carcinomas (HCC) (16). On the other hand, the altered microbiome of an obese mother is transmitted to the offspring at the time of birth (17). Wankhade et al. have shown that this altered gut microbiome in offspring of obese mothers in turn leads to metabolic changes in the offspring (18). Furthermore, several groups have shown that epigenetic changes that happen during the fetal development are passed on from parents to the offspring and modulate the risk for cancer (19–21). It is likely that these factors also play an important role in the increased risk for HCC in the offspring of obese mothers, besides the regulation of gene expression through specific microRNAs.

The current obesity pandemic will undoubtedly have a long-lasting impact on the health of future generations and given the epidemiological evidence, we will not see the full effect for many decades to come. Sun et al. have recently reported that maternal obesity increases the risk to develop HCC through microRNA mediated downregulation of *Acsl1* and *Aldh2*. In this meta-analysis we showed that the expression of these genes is not affected by maternal obesity in a number of studies. This highlights the importance to study other mechanisms of transmission. More than ever, we need to act and prevent harm to children that are born to obese mothers. We need to fully understand the mechanisms of risk transmission from mother to offspring in order to develop efficient treatments for mothers affected by obesity and their children.

## Author Contributions

BM, SL, and CT: conceptualization and writing. BM and SL: formal analysis. SL and CT: supervision, project administration, and funding acquisition. All authors have read and agreed to the published version of the manuscript.

## Funding

The Swiss National Science Foundation (Grant Number 182471), the Fondation Francis et Marie-France Minkoff, and the Leenaards Foundation (Grant Number 5489) funded this research.

## Conflict of Interest

The authors declare that the research was conducted in the absence of any commercial or financial relationships that could be construed as a potential conflict of interest.

## Publisher's Note

All claims expressed in this article are solely those of the authors and do not necessarily represent those of their affiliated organizations, or those of the publisher, the editors and the reviewers. Any product that may be evaluated in this article, or claim that may be made by its manufacturer, is not guaranteed or endorsed by the publisher.
